# Another Field of Usefulness

**Published:** 1900-08

**Authors:** 


### ANOTHER FIELD OF USEFULNESS.

THE *Journal* has watched with much pleasure and deep interest the valuable work done by members of the veterinary profession among the sister profession of human medicine. In Washington, Minnesota, Michigan, Pennsylvania, and other States veterinarians have entered the conventions of medical men to discuss sanitary problems, and have awakened a deeper interest in certain aspects of our own work. Medical men of years' standing, graduates of the older classes, and many of the graduates of to-day, have been sent forth to practise totally ignorant of the identity of a number of diseases in human and comparative pathology and their mode of propagation, development, and perpetuation. To enlighten medical men on these points is to interest them in all well-laid plans for the control of many infectious and contagious diseases, and is a work of untold value, and veterinarians should be alert and eager to disseminate knowledge in such channels, as it is sure to come back in a broader, more substantial recognition of our field of usefulness. The need of general knowledge and the teaching of food-inspection to medical students will promote a radical change in this field of our work, and we are hopeful of an early day's stand of all medical schools in making this important field of research a part of the curriculum. When medical men are better informed and their interest awakened, there will follow a more rapid education of the people and preventive medicine along veterinary sanitary lines, meat- and milk-inspection, etc., will receive an impetus of much import.

Joint-meetings of those engaged in human and comparative medicine are of great value. Veterinarians, becoming members of medical organizations, open up a wide field of usefulness. Veterinarians on State and municipal boards of health find golden opportunities for valuable work, and we urge our colleagues to join heartily in this movement.

Frequent contributions to medical journals on topics of mutual interest are sure to attract deserved attention and thus bring incalculable good to the progress of preventive medicine, a boon to suffering humanity, and a rich reward to those engaged in the field of comparative medicine.

